# Pregnancy outcomes following nonobstetric surgery during gestation: a nationwide population-based case-control study in Taiwan

**DOI:** 10.1186/s12884-018-2079-4

**Published:** 2018-11-26

**Authors:** Chia-Hung Yu, Shih-Feng Weng, Chung-Han Ho, Yi-Chen Chen, Jen-Yin Chen, Ying-Jen Chang, Jhi-Joung Wang, Ming-Ping Wu, Chin-Chen Chu

**Affiliations:** 1 Division of Women’s and Children’s Anesthesia, Department of Anesthesiology, Chi Mei Medical Center, 901 Zhonghua Road, Yongkang District, Tainan City, 710 Taiwan; 2 Department of Healthcare Administration and Medical Informatics, Kaohsiung Medical University, 100, Shih-Chuan 1st Road, Kaohsiung, 807 Taiwan; 3 Department of Medical Research, Chi Mei Medical Center, 901 Zhonghua Road, Yongkang District, Tainan City, 710 Taiwan; 4 Department of Pharmacy, Chia Nan University of Pharmacy and Science, Chi Mei Medical Center, 60, Sec. 1, Erren Rd., Rende Dist., Tainan City, 717 Taiwan; 5 Department of Senior Citizen Service Management, Chia Nan University of Pharmacy and Science, Chi Mei Medical Center, No.60, Sec. 1, Erren Rd., Rende Dist, Tainan City, 717 Taiwan; 6 Division of Urogynecology and Pelvic Floor Reconstruction, Department of Obstetrics and Gynecology, Chi Mei Medical Center, 901 Zhonghua Road, Yongkang District, Tainan City, 710 Taiwan; 7 Center of General Education, Chia Nan University of Pharmacy and Science, Chi Mei Medical Center, 60, Sec. 1, Erren Rd., Rende Dist.,, Tainan City, 717 Taiwan

**Keywords:** Pregnancy, Nonobstetric surgery, Abortion, Delivery outcome

## Abstract

**Background:**

Whether nonobstetric surgery during gestation is associated with a higher risk of spontaneous abortion or adverse delivery outcomes is still unclear.

**Methods:**

We performed a retrospective case-control study using a Longitudinal Health Insurance Database (LHID 2000) containing claim-data of 1 million randomly selected beneficiaries. We compared the incidences and estimated the adjusted odds ratios (aOR) with 95% confidence interval (95% CI) for spontaneous abortion, adverse delivery outcomes, cesarean delivery, and prolonged hospital stay to determine the risk of adverse outcomes in women who had nonobstetric surgery during gestation as compared to those who did not have any surgery during gestation.

**Results:**

After exclusion, we were left with 114,852 delivery and 3999 abortion cases in our study; and 462 (0.39%) of them had nonobstetric surgery under general or regional anesthesia during pregnancy. The leading surgeries were repair of cervical os (33.12%), appendectomy (17.32%), ovarian surgeries (13.64%), and fixation of fractured bone (8.01%).The risk of spontaneous abortion (4.23% vs. 2.43%, aOR:1.53; 95% CI: 1.01–2.31), antepartum hemorrhage (7.14% vs. 2.83%, aOR: 2.51; 95% CI: 1.74–3.61), pre-eclampsia/eclampsia (2.60% vs. 1.01%, aOR: 2.35; 95% CI: 1.30–4.23), gestational diabetes (2.38% vs. 0.69%, aOR: 3.12; 95% CI: 1.69–5.78), prematurity (9.06 vs. 4.90%, aOR: 3.31; 95% CI: 2.54–4.31), cesarean section (43.55% vs. 33.76%, aOR: 1.41; 95% CI: 1.17–1.71), and prolonged hospital stay (1.82% vs. 5.91%, aOR: 3.23; 95% CI: 2.16–4.83) were higher in those women who had nonobstetric surgery after adjusting for age and comorbidities.

**Conclusions:**

Nonobstetric surgery during gestation were associated with a higher risk of spontaneous abortion, adverse delivery outcomes, cesarean section, and prolonged hospital stay.

## Introduction

Nonobstetric surgeries, including those directly related to pregnancy (e.g., cerclage or ovarian cystectomy) and those which are un-related to pregnancy (e.g., appendectomy or surgery for bone fracture) [1], are performed in 0.75 to 2.0% of all pregnancies worldwide [2]. Surgery during pregnancy is associated with a higher incidence of postoperative adverse events, such as septicemia, pneumonia, urinary tract infection, and in-hospital mortality [3–5]. However, the American College of Obstetricians and Gynecologists’ Committee still concluded that a pregnant woman should never be denied an indicated surgery [6]. Patients with specific disease such as acute appendicitis during pregnancy, managed conservatively, had a higher risk of shock, peritonitis, and venous thrombosis than those managed surgically [5].

Most of the available scientific literature about this topic mainly focus on diagnosis, surgical management, and immediate maternal complications [3, 7–11]. Only few studies have reported information about abortion [12], fetal outcomes [13–15], and obstetric outcomes [13, 16] in women having nonobstetric surgery during pregnancy. Recently, a large scale British study analyzed the association of adverse obstetric outcomes in such patients [16]. However, some important adverse obstetric outcomes such as antepartum hemorrhage, premature rupture of membrane, pre-eclampsia/eclampsia, and fetal distress have still not been reported.

Besides, no similar studies are available on this topic in the Asian population. Different ethnic races may have different epidemiological profiles and perhaps different outcomes. Studies using big data from ethnicities other than Caucasian are warranted. Therefore, we aimed to assess the risk of miscarriage and adverse pregnancy outcomes following nonobstetric surgery during gestation by using a nationwide population-based database in Taiwan.

## Methods

### Ethical review

The present study was granted an exemption from a full ethical review by Chi Mei Hospital Institutional Review Board (Tainan, Taiwan, Chairperson Prof., Chung HsiHsing, Ethical Committee No. 10402-E04) on 12 February 2015. Since the study had a retrospective design and all types of personal identification in the database were encrypted to secure patient privacy, the committee also waived the need for obtaining informed patient consent.

### Database

Taiwan launched a single-payer National Health Insurance (NHI) program on March 1, 1995. The NHI offers comprehensive medical care coverage to all Taiwan residents. As of 2015, about 22.6 million (more than 99%) of Taiwan’s 22.96 million legal residents (citizens and noncitizens) were enrolled in this program. The National Health Research Dataset (NHIRD) provides encrypted patient identification numbers, gender, date of birth, dates of admission and discharge, the ICD-9-CM (International Classification of Diseases, Ninth Revision, Clinical Modification) codes of diagnoses and procedures, details of prescriptions, and costs covered and paid for by the NHI.

For longitudinal follow up of the pregnancy outcomes after nonobstetric surgery, we used the Longitudinal Health Insurance Database 2000 (LHID 2000), a sub-dataset of the NHIRD, which contains all claim data (from 1997 to 2013) of 1 million beneficiaries who were randomly selected from the system in 2000. All the registration and claim data of the sampled people constituted this longitudinal database, and their healthcare data is updated annually. There was no significant difference in age, gender, or average insured payroll-related premiums between the sample group and all enrollees. The LHID 2000 database was thus considered to have representative power of the national population [17].

### Study sample

**Fig. 1:**
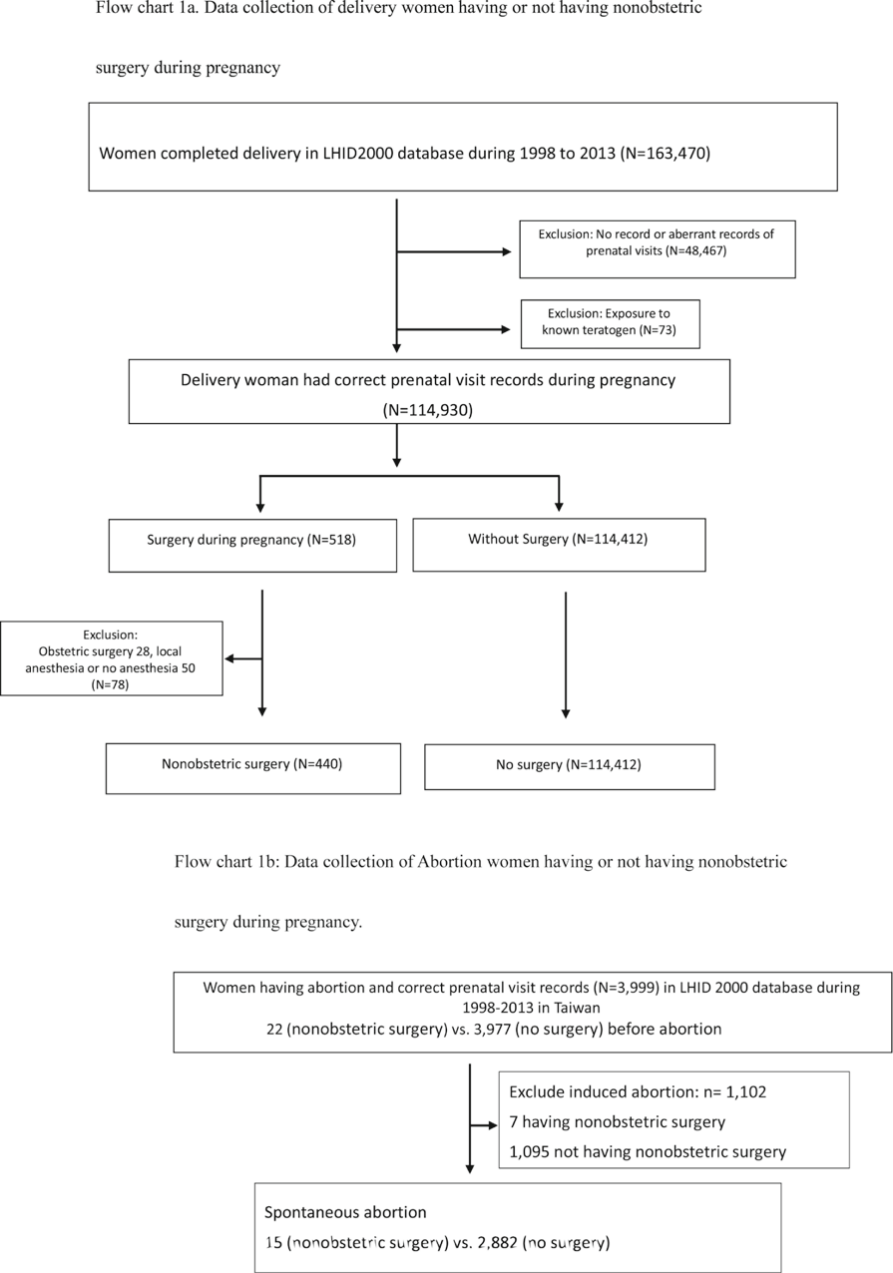
Flow charts of the creation of the study sample. **a** Delivery women having or not having nonobstetric surgery during pregnancy. **b** Abortion women having or not having nonobstetric surgery during pregnancy

We identified delivery event using the Diagnosis Related Groups code (DRG code 0373A, 0373C, 0371A or 0373B) and abortion by the International Classification of Diseases, 9th Revision, Clinical Modification (ICD-9 CM code 634-637). They were traced back whether having a nonobstetric surgery during pregnancy (Fig. 1a and b).

Nonobstetric surgery was defined as any surgery performed during pregnancy except, (i) fetal surgery, (ii) dilatation and curettage, (iii) surgery on gestational uterus body, and (iv) cesarean delivery. Surgery on adnexa or uterine cervix was still included as nonobstetric surgery, as classified in a previous research [7].

Nonobstetric surgeries were further subclassified into four main categories: (i) digestive system (ICD-9 procedure code: 42-54), including appendectomy, biliary surgery, and hemorrhoidectomy etc., (ii) female genital system (ICD-9 procedure code: 60-64), for example, repair of cervical os and ovarian surgery etc., (iii) musculoskeletal system (ICD-9 procedure code: 76-84), e.g., open reduction and internal fixation, and (iv) other systems. Anesthesia types were identified by payment code: general (96020C~96022C) or neuraxial anesthesia (96005C~96008C). Operations performed under local or no anesthesia were excluded from the study. In addition, patients who were ever exposed to known teratogenic factors, such as chemotherapy or radiotherapy, during gestation were also excluded from the study.

Further, we determined the trimester of pregnancy by the records of the prenatal visits (sequence code v41~50). Pregnant women who had no prenatal visits or with aberrant records were excluded from the study because of the difficulty in confirming the gestational age at which the nonobstetric surgery was performed. Delivery modes were identified based on diagnosis-related groups (DRG) and surgery codes from the NHI hospital discharge data and were categorized into vaginal delivery (DRG 0373A or 0373C) and cesarean section (DRG 0371Aor 0373B).

### Outcomes

We set the study outcomes as (i) abortion (ICD-9 code, 634~637), but induced abortion (635~636) was excluded, (ii) premature labor (ICD-9 code, 644), (iii) premature rupture of membrane (ICD-9 code, 658.1), (iv): Fetal distress (ICD-9 code, 656.3~4768.4), (v): antepartum hemorrhage (ICD-9 code, 641,762.0~1),(vi) pre-eclampsia/eclampsia (ICD-9 code, 642.4~642.7), (vii) gestational diabetes (ICD-9 code, 648.8), (viii) maternal death (ICD-9 code, 761.6, 798) during delivery admission, (ix) Cesarean section (diagnosis-related group code, 0371A or 0373B), and (x) Prolonged hospital stay, defined as upper quartile of length of stay plus 1.5 times the interquartile range.

### Statistical analysis

SAS 9.3.1 for Windows (SAS Institute, Inc., Cary, North Carolina, USA) was used for analysis. Continuous variables were presented as mean (SD) or median (inter-quartile range), and categorical variables were presented as number (percentile). The differences in baseline characteristics and comorbid variables between the two cohorts were evaluated using Student’s t test for continuous variables and Pearson χ2or Fisher’s exact tests for categorical variables. Significance was set at *p* < 0.01.

Logistic regression analyses were performed to assess the adjusted odds ratio with 95% confidence interval (CI) of adverse pregnancy outcomes between the two cohorts.

## Results

Between 1997 and 2013 in LHID 2000 dataset, we identified a total of 163,470 events of delivery. After exclusion of 48,467 events without any or with aberrant prenatal visit records and 73 events having teratogenic exposures, we were left with 114,930 deliveries. After exclusion of 78 cases which underwent obstetric-related surgeries under local or no anesthesia, there were 440 deliveries which underwent nonobstetric surgeries, and 114,412 deliveries which did not undergo any surgery during pregnancy (Fig. 1a). Moreover, we identified 3999 events of abortion, and 22 of them had nonobstetric surgery during pregnancy (Fig. 1b). Totally, there were 462 (0.39%) cases having nonobstetric surgery during gestation (Table 1).

**Table 1:** Demographic characteristics of pregnant women in the study populations as per LHID2000^a^ dataset from 1997 to 2013 in Taiwan

Characteristics	Nonobstetric surgery during pregnancy		*P*-value^b^
	No (*N* = 118,389)	Yes (*N* = 462)	
Delivery	114,412	440	
Abortion	3.977	22	
Age (Mean ± SD)	29.16 ± 4.89	29.86 ± 5.48	0.007
< 20	2810 (2.37)	16 (3.46)	< 0.001
20–29	59,426 (50.20)	189 (40.91)	
30–39	53,977 (45.59)	240 (51.95)	
≧ 40	2176 (1.84)	17 (3.68)	
Gestation			< 0.001
Singleton	116,904 (98.75)	442 (95.67)	
Multifetal	1485 (1.25)	20 (4.33)	
Comorbidities			
Diabetes mellitus	189 (0.16)	0 (0.00)	1.000
Hypertension	199 (0.17)	4 (0.87)	0.008
Ischemic heart disease	40 (0.03)	2 (0.65)	0.012
Renal disease	56 (0.05)	0 (0.00)	1.000

^a^*LHID2000* Longitudinal Health Insurance Database 2000, a sub–dataset contains all claim data of 1 million beneficiaries who were randomly selected from the system in year 2000

^b^Chi-square test or Fisher’s exact test when the expected counts were fewer than 5

Data are mean ± S.D. or *n* (%). Significant different if *p*-value < 0.01

Maternal characteristics of the study groups are shown in Table 1. The proportions of patients younger than 20 years and older than 30 years were higher in the surgical group as compared to that in the non-surgical group (*p* < 0.001). Moreover, the number of pregnant mothers with incidence of multi-fetal gestation was significantly higher in the surgical group as compared to that in the non-surgical group (4.33 vs. 1.25%, *p* < 0.001). Incidence of hypertension was also significantly higher in the surgical group than that in the non-surgical group (0.87 vs. 0.17%, *p* = 0.008).

Out of the 462 cases in which non-obstetric surgeries were performed, 50.22% of surgeries were performed during 2nd trimester, whereas 36.15 and 13.64% of the surgeries were performed in the 1st and 3rd trimesters, respectively. About 76.19% of the nonobstetric surgeries were non-emergency surgeries under the Taiwan NHI definition: waiting time less than 4 h from emergency room visit to operation. Moreover, surgeries were more frequently performed under regional anesthesia in contrast to that under general anesthesia (59.74 vs. 40.26%, *p* < 0.001). The leading nonobstetric surgery was repair of cervical os (33.12%), followed by appendectomy (17.32%), ovarian surgeries (13.64%), and bone fracture surgeries (8.01%) (Table 2).

**Table 2:** The trimester in which the nonobstetric surgery was performed during pregnancy, as per LHID2000^a^ dataset, 1997–2013, in Taiwan

Classification	*N* (%)
Surgeries	462 (100)
Trimester	
1st	167 (36.15%)
2nd	232 (50.22%)
3rd	63 (13.64%)
Emergency surgery	
Yes	110 (23.81)
No	352 (76.19)
Anesthesia	
GA	186 (40.26)
RA	276 (59.74)
Surgery	
Digestive system	115 (24.90)
Appendectomy	80 (17.32)
Hemorrhoidectomy	12 (2.60)
Biliary surgery	7 (1.52)
Others	16 (3.47)
Genital system	258 (55.85)
Repair of cervical os	153 (33.12)
Ovarian surgeries	63 (13.64)
Fallopian tube surgery	14 (3.03)
Others	28 (6.06)
Musculoskeletal system	40 (8.66)
Open reduction & internal fixation	37(8.01)
Others	3 (0.65)
Other systems	49 (10.61)

^a^*LHID2000* Longitudinal Health Insurance Database 2000, a sub–dataset containing all claims data of 1 million beneficiaries who were randomly selected from the system in year 2000

Among the 462 pregnancies with nonobstetric surgery during gestation, 440 pregnancies resulted in delivery while seven underwent induced abortion and 15 had a spontaneous abortion. The spontaneous abortion rate was higher in patients who underwent nonobstetric surgery in contrast to that in those without surgery (4.23 vs. 2.43%, *p* = 0.002). Incidence of adverse delivery events, such as premature rupture of membrane (5.63 vs. 4.33%, *p* = 0.17), fetal distress (2.16 vs. 2.64%, *p* = 0.523) and maternal death (0.23 vs. 0.06%, *p* = 0.244), were similar in these two groups (Table 3). However, patients who underwent nonobstetric surgery had a higher incidence of antepartum hemorrhage (7.14 vs. 2.83%, *p* < 0.001), pre-eclampsia/eclampsia (2.60 vs. 1.01%, *p* = 0.003), gestational diabetes (2.38 vs. 0.69%, *p* < 0.001), prematurity (9.06 vs. 4.90%, *p* = 0.001), and cesarean section (43.55 vs. 33.76%, *p* < 0.001) as compared with that in patients without any surgery. In addition, length of hospital stay for delivery was also longer in those who underwent nonobstetric surgery. (4.72 ± 4.21 vs. 3.78 ± 3.09 days, *p* = 0.004) (Table 3).

**Table 3:** The incidence, crude and adjusted odds ratio of abortion and adverse delivery outcomes in study population as per LIHD2000 dataset in Taiwan, 1997–2013

Outcome	Nonobstetric surgery during pregnancy		*P*-value	Crude OR (95% CI)^a^	Adjusted OR (95% CI)^b^
Total	No (*N* = 118,389)	Yes (*N* = 462)			
Abortion	3997 (3.36%)	22 (4.76)	0.002		
Spontaneous	2882 (2.43)	15 (4.23)		1.57 (1.04–2.36)	1.53 (1.01~2.31)
Induced	1095 (0.92)	7 (2.28)			
Delivery	114,412 (96.64)	440 (95.24)			
PROM^c^	5122 (4.33)	26 (5.63)	0.170	1.37 (0.92–2.04)	1.36 (0.91–2.02)
Fetal distress	3127 (2.64)	10 (2.16)	0.5234	0.84 (0.45–1.58)	0.83 (0.44–1.56)
Antepartum hemorrhage	3355 (2.83)	33 (7.14)	< 0.001	2.63 (1.83–3.77)	2.51 (1.74–3.61)
Pre-eclampsia/Eclampsia	1197 (1.01)	12 (2.60)	0.003	2.67 (1.50–4.74)	2.35 (1.30–4.23)
Gestational diabetes	811 (0.69)	11(2.38)	< 0.001	3.61 (1.97–6.59)	3.12 (1.69–5.78)
Prematurity	5064 (4.90)	36 (9.06)	0.001	3.43 (2.63–4.46)	3.31 (2.54–4.31)
Cesarean section	38,624 (33.76)	125 (43.55)	< 0.001	1.48 (1.22–1.79)	1.41 (1.17–1.71)
Maternal death	72 (0.06)	1(0.23)	0.244	3.62 (0.50–26.09)	3.14 (0.42–23.19)
^d^Prolonged hospital stays	2078 (1.82)	26 (5.91)	< 0.001	3.40 (2.28–5.06)	3.23 (2.16–4.83)
Hospital stay (days)					
Mean ± SD	3.78 ± 3.09	4.72 ± 4.21	< 0.001		
Median (Q1–Q4)	3 (2–5)	4 (3–5)			

Data are number (%), except hospital stay (days)

^a^*OR* odds ratio, ^b^*Adjusted OR* adjusted by age and comorbidities

^c^*PROM* premature rupture of membrane, ^d^Prolonged hospital stay was defined as the upper quartile of length of stay plus 1.5 times the interquartile range

Women had nonobstetric surgery during pregnancy were associated with higher crude odds ratio of adverse outcomes, except PROM, fetal distress and maternal death during delivery. Moreover, after adjusting for age and comorbidities, pregnancies with nonobstetric surgery were still associated with a significantly higher risk of premature labor (aOR, 3.31; 95% CI, 2.54–4.31), antepartum hemorrhage (aOR, 2.51; 95% CI, 1.74–3.61), pre-eclampsia/eclampsia (aOR, 2.35; 95% CI, 1.30–4.23), gestational diabetes (aOR, 3.12; 95% CI, 1.69~5.78), cesarean section delivery (aOR, 1.41; 95% CI, 1.17–1.71), prolonged hospital stay (aOR, 3.23; 95% CI, 2.16~4.83) as compared to that in pregnancies without any surgical intervention.

The risk factors for premature labor in cases who underwent nonobstetric surgery during pregnancy were further analyzed (Table 4). Deliveries with nonobstetric surgery in the 3rd trimester had a 3.79-fold (95% CI, 1.20–11.96) odds as compared to that with surgery in the 1st trimester. Among the specific category of nonobstetric surgeries, only musculoskeletal surgery was associated with a higher risk of prematurity. Moreover, emergency cases and general anesthesia were not associated with a significantly higher risk of premature labor in contrast to non-emergency cases (aOR, 1.32; 95% CI, 0.65–2.69) or regional anesthesia (aOR, 1.78; 95% CI, 0.89–3.57).

**Table 4:** The odds ratio of premature labor in pregnant women having nonobstetric surgery as per LHID2000^a^ dataset, 1997–2013, Taiwan

	Prematurity		Crude OR^b^ (95% CI)	Adjusted OR^c^ (95% CI)
	No = 374	Yes = 66		
Age^d^ (Mean ± SD)	29.65 ± 5.61	30.86 ± 4.88	1.48 (0.93–2.35)	1.25 (0.70–2.23)
Trimester				
1st	143 (38.24)	12 (18.18)	1.00	1.00
2nd	185 (49.47)	40 (60.61)	2.58 (1.30–5.09)	2.04 (0.93–4.50)
3rd	46 (12.30)	14 (21.21)	3.63 (1.57–8.40)	4.10 (1.62–10.39)
Emergency surgery				
No	285 (76.20)	50 (75.76)	1.00	1.00
Yes	89 (23.80)	16 (24.24)	1.02 (0.56–1.89)	1.32 (0.65–2.69)
Anesthesia				
GA	159 (42.51)	15 (22.73)	1.00	1.00
RA	215 (57.49)	51 (77.27)	2.51 (1.36–4.63)	1.78 (0.89–3.57)
Surgical procedure				
Genital organ	196 (52.41)	49 (74.24)	9.00 (1.20–67.27)	5.70 (0.75–43.56)
GI	97 (25.94)	14 (21.21)	5.20 (0.66–40.95)	4.39 (0.55–34.98)
Musculoskeletal	36 (9.63)	1 (1.52)	1.00	1.00
Others	45 (12.03)	2 (3.03)	1.60 (0.14–18.36)	1.23 (0.10–14.58)

^a^*LHID 2000* Longitudinal Health Insurance Database 2000 contains claims data of 1 million beneficiaries who were randomly selected from the system in year 2000

^b^*OR* odds ratio

^c^Adjusted by age, comorbidities, and all obstetric conditions

^d^Per 10-year increase

## Discussion

Using the database of 1 million randomly selected beneficiaries in Taiwan from 1997 to 2013, we noted a higher incidence and risk of spontaneous abortion, premature labor, antepartum hemorrhage, pre-eclampsia/eclampsia, gestational diabetes, and cesarean section in pregnancies with a nonobstetric surgery during their gestation in contrast to that with pregnancies without any surgery, after adjusting for age and any pre-existing comorbidities. This study is the first Asian population-based study to evaluate the pregnancy outcomes of women undergoing nonobstetric surgeries during gestation, and some of the findings are novel i.e. increased risk of antepartum hemorrhage, pre-eclampsia/eclampsia, and gestation diabetes in pregnant women who have undergone nonobstetric surgery during pregnancy.

Our analysis was based on a 17-year large-scale longitudinal database, a case control study design, and multivariable adjustments. Most previous studies were either small scale [8, 18, 19] or performed decades ago [13, 14, 19]. Although, recently, a large-scale British study [16] has been published on this topic, no similar study has been done in the Asian population; ethnic disparity may have different epidemiological profiles and diverse outcomes.

The incidence of nonobstetric surgery during pregnancy was reported to be between 0.75 and 2.0% [2]. In our study, the incidence (0.39%) is lower than 2%, which is lower as compared to that in other large-scale retrospective studies from Sweden (0.75%,1973–1981) [13] and Britain (0.73%, 2002–2012) [16]. We speculate that the racial disparity, life-style, and social-economic factors may all have contributed to this difference.

Our results revealed that the leading types of nonobstetric surgeries during pregnancy were repair of cervical os (33.1%), followed by appendectomy (17.3%), ovarian surgeries (13.6%), and repair of fractured bone (8.0%) in Asian women. Some studies excluded all surgeries on gravid uterus and fetus from nonobstetric surgeries [16]; however, some excluded only surgeries on the fetus during pregnancy from nonobstetric surgeries [7]. We followed the latter definition.

A meta-analysis of 54 studies reported that the abortion rate was 5.76% in pregnancies with nonobstetric surgeries during gestation; however, this number is difficult to interpret since matched controls were not available in that study [7]. In the recent large-scale British study, although only miscarriage during hospitalization was included, it revealed a higher spontaneous abortion rate (6.6 vs. 5.8%) in patients having nonobstetric surgery. Similarly, we also found a higher rate and risks of spontaneous abortion (4.23 vs. 2.43%, aOR 1.53, 95% CI: 1.01~2.31) in Asian women who underwent nonobstetric surgery during gestation. In contrast to the British study, we counted all abortions (both in- and out-patient services) after the nonobstetric surgery. Besides, we observed that the abortion rate was lower in Asians as compared to that in Caucasians in both groups of pregnant women i.e. those who underwent or those who did not require any nonobstetric surgery.

In previous studies, the incidence of prematurity after nonobstetric surgery was estimated to be around 3.5~8.2% [7]. In our study, risk factors associated with prematurity were analyzed further, including maternal age, trimester when the surgery was performed, emergency or elective surgery, type of surgical procedure and the choice of anesthesia. In the present study, it was observed that surgeries performed in the third trimester were associated with an increased risk of premature labor further as compared to those performed in the first and second trimester. The result is compatible with what had been observed in the study in 2001 [20].

Asides from abortion and preterm delivery, we also found increased risks of cesarean section and long hospital stays. These were similar to previous reports [16, 21, 22]. Taken together, increased abortion, preterm delivery, cesarean section rate and longer in-patient stay are the common and most reliable findings in the existing literature.

With the improvement in anesthetic techniques, perioperative care and surgical techniques, maternal death has decreased to around 0.004~0.025% [7, 11, 16, 23]. In our study, we found a relatively higher mortality rate (0.064%, 72 vs. 1 in the non-surgical and surgical group, respectively) than previous reports. Especially, the mortality in nonobstetric surgery patients yields a high mortality rate (0.23%), however, the wide range of 95% CI (0.42~23.19) indicated that our nonobstetric surgical group is not large enough to analyze the rare event of mortality. We suspected the large number of patients without prenatal visits (46,467) excluded from our study might distort the current observed results. However, the large-scale British study demonstrated a 4.67-fold increase in mortality risk (95% CI, 1.79–8.93) in the pregnant women with surgical procedure during gestation [16].

The mechanisms of our novel findings of increased risk of antepartum hemorrhage, pre-eclampsia/eclampsia and gestational diabetes were not determined, because this is a retrospective database study. However, there were some possible explanations. Firstly, the inflammatory reaction or scar formation after nonobstetric surgery may further increase the complexity of anatomy of abdominal organs and pregnancy physiology [24]. Secondly, these adverse delivery outcomes may be delayed sequelae of nonobstetric surgery, which were reportedly associated with increased risks of in-hospital pneumonia, septicemia, and postoperative bleeding [3]. Thirdly, some of the nonobstetric surgical diseases persist as long term systemic disease even after surgical treatment, e.g. patients with Crohn disease [25].

### Limitations

Our findings need to be interpreted in the context of some inherent limitations of a claim database. Firstly, identifying these cases, associated comorbid disorders, and adverse outcomes was dependent upon the accuracy of the ICD-9-CM coding. Although, these codes were reviewed and validated by auditors of medical records for the insurance system to ensure the accuracy of the claims, incorrect coding might still exist. Secondly, due to lack of reporting about fetal gestational age, we had to estimate the trimester of pregnancy using the records of prenatal visits. Therefore, we had to exclude 48,467 (29.65%) pregnant women who did not have any or with aberrant prenatal visit records from our study. This might result in an equally distribution of adverse outcomes in the two groups of patients, and caused an under- or over-estimate of association of some adverse outcomes. Thirdly, some important individual data about lifestyle behaviors, such as smoking and alcohol drinking habits, body mass index, and the severity of comorbid conditions were not available in the NHIRD. Thus, we could not adjust for these variables as contributing factors. Fourthly, fetal information was lacking in the database; therefore, we could not analyze the fetal outcome, any congenital anomaly, still birth, or fetal death. This also restricted further discussion about the teratogenic effect associated with the surgeries or anesthesia. Lastly, repeat procedures in duplicate patients were excluded. This reduced the total case number to be analyzed; however, these patients might have different outcomes.

## Conclusions

Nonobstetric surgery during pregnancy was associated with an increased risk of miscarriage, premature labor, and higher cesarean section rate. Further large-scale prospective studies with detailed fetal information may help to clarify the effects of nonobstetric surgery on parturient women and the newborns.

## Abbreviations

95% CI: 95% confidence interval
aOR: adjusted odds ratio
ICD-9-CM: International Classification of Diseases, Ninth Revision, Clinical Modification
LHID 2000: Longitudinal Health Insurance Database 2000
NHI: National Health Insurance
OR: Odds ratio
